# Silent Contamination: The State of the Art, Knowledge Gaps, and a Preliminary Risk Assessment of Tire Particles in Urban Parks

**DOI:** 10.3390/toxics11050445

**Published:** 2023-05-09

**Authors:** Lorenzo Federico, Andrea Masseroni, Cristiana Rizzi, Sara Villa

**Affiliations:** Department of Earth and Environmental Sciences DISAT, University of Milano-Bicocca, Piazza della Scienza 1, 20126 Milano, Italy; l.federico8@campus.unimib.it (L.F.); a.masseroni2@campus.unimib.it (A.M.); cristiana.rizzi@unimib.it (C.R.)

**Keywords:** tire particles, soil pollution, ecotoxicology, environmental risk assessment

## Abstract

Tire particles (TPs) are one of the main emission sources of micro- and nano-plastics into the environment. Although most TPs are deposited in the soil or in the sediments of freshwater and although they have been demonstrated to accumulate in organisms, most research has focused on the toxicity of leachate, neglecting the potential effects of particles and their ecotoxicological impact on the environment. In addition, studies have focused on the impact on aquatic systems and there are many gaps in the biological and ecotoxicological information on the possible harmful effects of the particles on edaphic fauna, despite the soil ecosystem becoming a large plastic sink. The aim of the present study is to review the environmental contamination of TPs, paying particular attention to the composition and degradation of tires (I), transport and deposition in different environments, especially in soil (II), the toxicological effects on edaphic fauna (III), potential markers and detection in environmental samples for monitoring (IV), preliminary risk characterization, using Forlanini Urban Park, Milan (Italy), as an example of an urban park (V), and risk mitigation measures as possible future proposals for sustainability (VI).

## 1. Introduction

Highway and road runoff are one of the main sources of pollution of the environment [1], contributing to the release of a plethora of contaminants such as hydrocarbons (HCs), heavy metals (HMs), micro-plastics or nano-plastics (MPs and NPs), and airborne particulate matter (PM) [2]. However, road traffic contributes to the release of insidious and silent contaminants into the environment, such as MPs produced by tire tread wear: tire particles (TPs).

This contamination was understated for a long time due to a poor consideration of synthetic rubbers in the definition of plastic; in fact, for the International Organization for Standardization (ISO), plastic is defined as a “material which contains as an essential ingredient a high polymer and which, at some point in its transformation into finished products, can be shaped by flow” [3]. Except for thermoplastics and thermosets, elastomers such as rubbers, which compose the tread of tires, have been excluded from this definition. However, Hartmann et al. [4] elaborated an inclusive plastic debris classification, including elastomers (synthetic rubber), heavily modified natural polymers or vulcanized natural rubber, and inorganic and hybrid polymers contained in the tire rubber. Therefore, tire wear particles are considered one of the main hidden sources of MPs and NPs which will require more insights in the future. [5,6,7,8,9]. 

Recent studies have shown that TPs could be responsible for about 40% by weight of the total amount of MPs in rivers in Europe, with concentrations up to 179 mg/L in sediments flowing from rivers [10]. TPs are furthermore responsible for more than 50% of the contamination of the soil, as shown by the fraction content in the urban and rural soils of China [11]. 

Globally, it has been estimated that more than 6 million tonnes of TPs per year are released, of which 1 million tonnes per year are from the European Union and the United States [12,13,14]. In general, TPs are found in every environment, especially in the soil ecosystem, which is strongly polluted by TPs with an estimated annual release of plastic from 4 to 23 times higher than that in the ocean [15]. Only 12% of TPs eventually reach the surface of the water through sewers; despite this, the large environmental load of TPs in the soil is underestimated and deserves to be investigated. The crucial role of particles produced by tire degradation and their human health impact have been well documented in recent studies [16,17,18,19,20], while others have been concerned with the potential environmental risks associated with using tire fill material [21,22]. However, most of these studies have focused only on the chemical leachate derived from the TPs and most of the observed effects have rarely come down to the molecular level. 

The aim of this review is to sift through, especially for the soil ecosystem, the current knowledge about the environmental contamination of TPs, paying particular attention to the chemical composition and degradation processes of tires (I), the transport and deposition in the environment according to their size and density (II), the potential markers and detectors in environmental samples for monitoring (III), the toxicological effects in edaphic organisms (VI), a preliminary risk characterization, paying particular attention to the TPs emission in Forlanini Park, Milan (Italy), as a sampling area (V), and some risk mitigation measures as possible future proposals for sustainability (VI).

## 2. Materials and Methods

This review was drafted by searching the literature for keywords such as “tire particles”, “soil”, “ecotoxicology”, “risk assessment”, and “sustainability”. The use of datasets such as Web of Science or Minerva and Prometheus (University of Milano-Bicocca) was utilized for this research. For a coherent review supported by scientific data, recent (less than ten years) and old (more than ten years) articles and reviews were considered for a general overview of the composition, fate, and environmental markers of TPs. A further criterion for the literature selection was applied for the ecotoxicological effect section, skimming the time frame and selecting only suitable items from 2020 until January 2023 in order to report recent studies. Overall, about 119 articles of the 137 open-access articles related to TPs in soil were selected. Only peer-reviewed articles were included.

## 3. Definition and Chemical Composition of Tire and Road Wear Particles

TPs occur due to mechanical friction between tires and the road surface during driving, acceleration, or braking, and the heat generated alters the original chemical composition of the wear particles [23,24]. Factors such as the climate, the type of tire, the road surface, the nature of the contact, the speed, the weight of the vehicle, and the driving style could affect their production [13,25,26]. It is estimated that an average car tire lasts between 20–50,000 km before wearing out, releasing about 10–30% of the tread rubber into the environment (at least 1–2 kg) [27].

When they come into contact with asphalt, the TPs undergo a morphological and dimensional change due to the incorporation of the road surface material and the increasing particle core size [28,29]. These aggregates are defined as tire and road wear particles (TRWPs), which have a coating of 10–50% by volume [24] and significantly change their density from 1.13–1.16 g/m^3^ to 1.5–2.2 g/cm^3^ [30,31]. The most abundant percentage mass (about 90–95%) of TRWPs is made up of heavy particles defined as ‘coarse particles’ [32], which are deposited on soil, sediment, or freshwater environments and not suspended in the air, while small amounts (maximum 10% of the total mass) are made up of a more volatile fraction and emitted into the air, and they are defined as ‘fine particles’ [28,33,34]. Particle size distribution and fractionation during transport into the environment is not currently known but could range from 10 nm to <5 mm [6,7,8,9,35,36]. 

**Figure 1:**
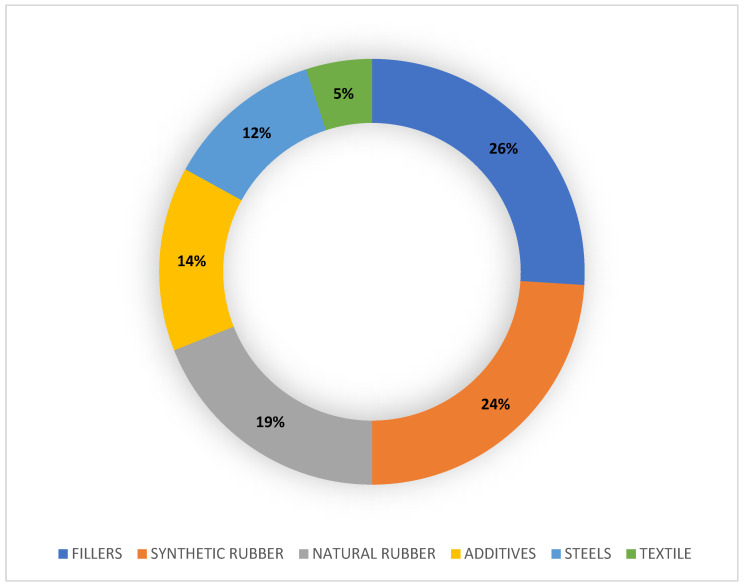
Donut chart relating to the percentage average composition of car tire treads. The data come from the literature [37,38].

The average percentages of a car tire’s composition are shown in Figure 1. The tread of a tire has a wide variety of chemical compounds, mainly:

- Rubbers: Components typically consist of blends of styrene-butadiene rubber (SBR), such as polybutadiene (PBD), and isoprene rubber (IR), the forerunner of natural rubber (NR), mixed with carbon black or silica (as a reinforcing agent/filler), oils (as softeners and extenders), and curing chemicals. In the past, tires were made only of natural rubber, such as that extracted from the Brazilian rubber tree *Hevea brasiliensis* [39]; nowadays, also for ecological reasons, a mixture of natural and synthetic rubbers is used. Such SBRs describe families of synthetic rubbers derived from emulsion-polymerized (E-SBR, more widely used) or solution-polymerized (S-SBR) styrene and butadiene. The styrene/butadiene ratio affects the properties of the rubber: if the styrene content is high, the rubbers are harder and less rubbery. Normally, 23.5% of the rubber consists of styrene and the remaining 76.5% consists of butadiene [40].
- Organic chemicals: Benzoic acid (BZA) and N-nitrosodiphenylamine (NDphA) are burning retarders and slow down the vulcanization process [34]. Diamines and waxes are also used as anti-degradants by oxidizing agents (oxygen or ozone) and by heat [41,42]. Studies on TRWPs leachate confirmed that they are a potential source of benzothiazoles (BTs), as accelerators of vulcanization, and 1-octanethiol (1-OT) [43], phthalates (PTEs), additives, such as bisphenol A (BSA), and polycyclic aromatic hydrocarbons (PAHs), such as benzo-**γ**-perylene, fluorene, benzo-**α**-pyrene, benzo-**β**-fluoranthene, phenanthrene, benzo-**κ**-fluoranthene, pyrene, anthracene, and fluoranthene [35,44,45,46,47]. Other substances released in leachate are N-(1,3-dimethylbutyl)-N′-phenyl-p-phenylenediamine (6-PPD) and its ozonation product 6-PPD-quinone [48], used as stabilizing additives, hexa(methoxymethyl)melamine (HMMM) [49,50,51], and N,N′-diphenylguanidine (DPG), an accelerator of vulcanization [14,52].
- Heavy metals (HM): Trace elements such as Zn, Al, Fe, Cd, Cr, Ni, Hg, and Cu are present on TRWPs [43]; consequently, tire wear contributes to the release of HMs into the environment. About 1% of zinc oxide (ZnO) is used as a catalyst to vulcanize the rubber mixtures, transforming them into highly elastic matter; consequently, TRWPs are considered to be among the main sources of Zn in the environment [53]. Other accelerators are sulfur, sulphenamides, and thiazoles [34].
- Fillers: Most of the components of a tire tread consist of fillers, mainly black carbon (22–40%) finely pulverized by incomplete combustion and added for making the tire resistant to UV rays. In recent years, carbon is sometimes replaced by nanometer glass spheres of silica, which gives the tread strong adhesion and resistance to tearing, heat, and ageing [13].

## 4. Environmental Fate of TRWPs

### 4.1. Transport and Deposition Pathways

**Figure 2:**
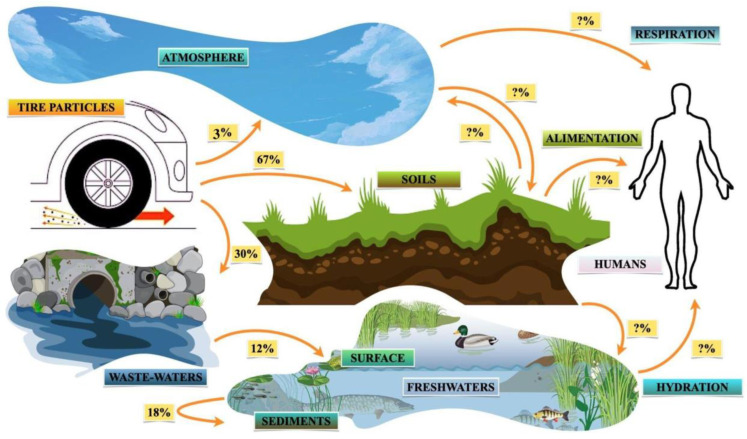
Pathways of the transport, deposition, and fate of TRWPs in environments such as soils, the atmosphere, wastewaters, sediments, and freshwater [13,60,61]. Most of the TRWPs accumulate in the topsoil (67%), while during storm events, they end up accumulating in wastewater (30%) through road surface sewers, where they are transported and accumulate in freshwater such as rivers and lakes, settling in sediments (18%) or remaining on the surface (12%); only a small fraction of the ‘fine particles’ end up in the atmosphere (3%), but they are also the most unstable, as they can settle after a long time and be resuspended or not at all. The pathways of accumulation in organisms through respiration, feeding, or drinking are potentially viable, which makes these TRWPs particularly dangerous and treacherous.

Once produced, TRWPs deposited on roads can be mobilized and transported by wind, traffic-induced air currents or washed away by rainwater action to other compartments, such as topsoil, air, waste waters, sediment, water surfaces, or other road sections [24,54]. They may potentially drift, accumulate, aggregate, persist, leach, or degrade, affecting the stability of the exposed ecosystems such as those reported in Figure 2 [32,55].

Transport distances depend on particle size and density; coarse particles tend to settle very close to the roadside, within 30 m [34], while fine particles can remain suspended in the air for a long time before settling over many meters [1,2]. Transport of TRWPs can also be influenced by aggregation events both with natural particulates (homo-aggregation) and anthropic particulates (hetero-aggregation) [56,57,58,59]. Most of the TRWPs produced accumulate in the soil (about 67%), while the remainder accumulates in the air (3–5%) and in wastewater treatment systems (30%), where they settle in the purification sludge, are transported in freshwater, bioaccumulate, or biodegrade [13]. Only 12% of TRWPs eventually reach the surface of the water through sewers [35], while about 18–22% settle in sediment [60,61]. TRWPs can also bioaccumulate in soil organisms through the food web [62,63,64]. 

#### 4.1.1. Soil

Soil is the main ecosystem affected by TRWPs contamination. These particles are dispersed from the road surface, where the concentrations are between 0.7 and 210 g/kg d.w. [65,66,67]; similarly, coarse TRWPs concentrations in the soil range from 0.6 to 117 g/kg d.w. [66,68,69,70]. Soil concentrations of TRWPs decrease rapidly with the increase in the distance from the road, with a reduction of more than 80% at a 30 m distance from the road [66,69] and it decreased by 30–40% at a 10–30 cm depth in the soil [66], but the estimated concentrations depend on the type of environmental marker used for detecting these particles and the type of sampling carried out whether it was conducted in-depth or adjacent to busy roads. In addition, the distribution can vary according to various climatic (wind, water, and UV) and anthropic (vehicle traffic) variables; as degradation in the soil is slow, it is possible that they are resuspended in the air by wind erosion or washed away by runoff [69]. 

#### 4.1.2. Air

The finest particulates, with a diameter generally greater than a micrometer, are emitted directly into the air [71]. These have much longer residence times in the atmosphere than coarse particles due to their low specific weight and reduced density. In addition, once deposited even many kilometers away, they can be resuspended in the atmosphere by wind or the air turbulence of vehicular traffic [71]. The road air can contain about 0.4–11 µg/m^3^ of TRWPs [65,66,69,70,72], but estimated concentrations are a function of the sampling distance: in general, the concentration of fine TRWPs increases by 40–50% at about 18 m away from the roadside [66]. The scattering distance depends on wind action, particle size and density, vehicular traffic, and even temperature, ranging from a maximum distance of 30 m [69] to a distance of 86 m from the road [73]. The particle size distribution is variable: the dominant size is between 2.5–10 µm (PM10), while the portion below 2.5 (PM2.5) constitutes only 0.68% in reference to the mass [24,74]. 

#### 4.1.3. Sewer Systems, Freshwater, and Sediments

A major source of runoff from the road surface, after wind action, is rainwater, which contributes to the transport of TRWPs into the aquatic environment. In sewage systems connected to urban roads, the estimated concentration ranges from 0.3 to 179 mg/L [34,64,65,75]. In rivers, the concentrations during storm events range from 0.09 to 3.6 mg/L, while concentrations under dry conditions are below the limits of detection [65,76]. In sediments, TRWP concentrations are between 0.3 to 155 g/kg d.w., with the greatest concentration being observed in heavily trafficked areas [16,34,64]; however, some of the TRWP concentrations in sediments reported in the literature are based on the use of benzothiazoles (BTs) as a marker, which, being soluble in water, could underestimate the actual amount of TPs accumulating in the sediment [77]. 

Table 1 reports and summarizes the TRWP concentrations in different ecosystems.

### 4.2. Degradation Processes

**Figure 3:**
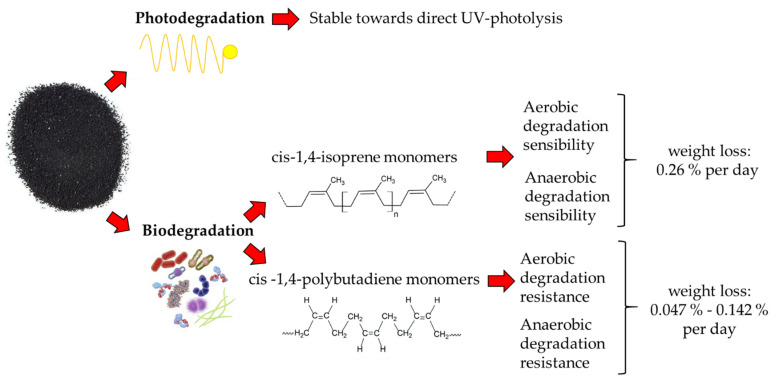
Diagram relating to the main degradative processes in which TRWPs are involved [32,78,79,80]. The photodegradation processes of rubbers are minimal and do not provide quantitative information, while biodegradation processes depend on the type of monomer involved (cis-1,4-isoprene or cis -1,4-polybutadiene) and the laboratory conditions.

The degradation of TRWPs (Figure 3), which depends on the susceptibility of photodegradation and biodegradation processes [32], presents a degradation rate of 0.15% per day [69]. 

Data relating to the photodegradation processes of rubbers are minimal and do not provide quantitative information [78]. Furthermore, the polymeric double bonds of rubbers can only be lysed by high-energy UV radiation (UV–C, 100–280 nm) and, consequently, are considered stable to direct UV photolysis [79]. Embrittlement due to UV radiation in the range of 290–400 nm is also prevented by protective agents such as antiozonants and antithermoxidants [79]. Concerning to biodegradation processes, cis-1,4-isoprene monomers, typical of natural and synthetic isoprene rubbers, are among the most sensitive to aerobic and anaerobic biodegradation, with a weight loss of about 0.26% per day [32,80]. This is because, during the vulcanization processes, the polyisoprene chains are covalently linked via sulfide bridges, which can be severed by aerobic and anaerobic microbes and modified, with an increase in hydrophilicity and unsaturated bonds [79]. Cis-1,4-polybutadiene monomers, typical of butadiene and styrene–butadiene rubbers, are resistant to biotic and abiotic oxidation, with a loss of 0.047% or 0.142% per day, respectively, depending on the absence or presence of styrene [32]. It has been demonstrated that *Nocardia* spp. 835A-Rc can degrade pneumatic dust under laboratory conditions [81]. Complete biodegradation of TRWPs is believed to be inhibited by the presence of co-formulates, such as Zn salts, BT, and 6-PPD [79,81]. 

### 4.3. Environmental Markers of TRWPs

For detecting TRWP pollution in soils and other ecosystems, different chemical markers can be used, as reported in Table 2. 

Elastomers such as styrene–butadiene (SBR) or natural rubber (NR) can be used as a marker to identify TRWPs in different environments [34,66,69,70]; indeed, 70% and 75% of the SBR and NR produced globally are totally employed in the production of tires, respectively [42]. 

BTs, used as vulcanization accelerators in tread manufacturing processes [77], are additional environmental markers for monitoring TRWPs in soil or sediment [34,64,65,73,76,83]. The main BTs include 24MoBT (2-(4-morpholinyl) benzothiazole), HOBT (2-hydroxybenzothiazole), and NCBA (Ncyclohexyl-2-benzothiazolamine); however, the use of BTs in industrial processes has decreased in less than two centuries [65]; in addition, BTs detected in the environment could have different origins, such as antifreeze products [34]. 

One of the most used environmental markers in the identification of TRWPs is certainly Zn, coming from the ZnO used as a catalyst in the tread vulcanization processes [34,53,84,85,86]. Despite its wide use, Zn appears to be the most generalist environmental marker of TRWPs, with it having different emission sources and being soluble in particulates, returning less precise vertical and horizontal content gradients, which may underestimate the real concentration of TRWPs in the environment [84]. Although Zn may appear to be a generic marker, methods have been developed to quantify extractable organic Zn as a marker of TRWPs in various environmental matrices [84]. 

In general, the detection of TRWPs can be performed by thermal extraction desorption gas chromatography–mass spectrometry (TED-GC/MS), quantifying the mass of MPs in soil samples produced after the thermal degradation of SBR, or by density separation processes. Zn can be quantified by inductively coupled plasma–optical emission spectroscopy (ICP-OES) after acid digestion of soil samples with hydrofluoric (HF) or nitric (HNO3) acid [84]. Spectroscopic methods are not recommended, because added carbon black causes total absorbance or fluorescence interferences [14]; however, Workek et al. [25] developed a spectroscopic method which combined Fourier transform infrared spectroscopy (FTIR) with a Raman spectrometer, capable of analyzing particles below 10 µm, to identify synthetic rubber.

In any case, it is legitimate to underline once again that environmental markers for the detection of TRWPs have limitations; in fact, some of these chemical markers are subject once leached to seizure or degradation, such as BT [38], which could lead to underestimation, but, at the same time, different emission sources could lead to an overestimation of the concentrations of these environmental markers of TRWPs [34].

## 5. Toxicity Effects on Edaphic Fauna and Plants

TRWPs have demonstrated important and often underestimated toxic effects and are starting to be an important stressor for ecotoxicological investigations [87,88]. In the literature, most of the studies conducted on TRWPs have focused on the leachate’s effects, mainly in aquatic environments [14,17,43,89,90,91]; only a few studies have investigated the effect of particles, despite previous studies showing that particles can be as toxic as the leachate [43,68,87,92,93,94]. Furthermore, studies on TRWP toxicity conducted in soil are still in the infancy stage and require more attention.

As reported by Khan et al. [94], a different toxicological trend of the particulate and of the leachate could not be excluded, perhaps following a dose-dependent trend in the first case and a non-sigmoidal trend in the other and suggesting an important factor of danger in the ingestion of particles and the release of chemicals into the digestive tract. 

To date, a proper risk assessment of TRWPs is challenging due to a lack of data. In this regard, it should be essential to draw up a repeatable and comparable experimental design, which takes into account the effects of tire particles as well as leachate, in order to provide more realistic ecological data. One of the main causes of this scarce and non-standardized data plethora is probably due to the lack of attention paid in the past to soils. 

Currently, in the literature, in addition to particles, the main factors responsible for the toxicity of TRWPs are co-formulates (PAHs, BTs, and HMs), and their overall toxicity depends on the size and density of the particles themselves, their composition, their aging, and the model organisms used during the experimental tests. Below, the major soil model organisms and plants exposed to TRWPs are reviewed (Table 3).

### 5.1. Earthworms

Regarding earthworms, *Enchytraeus crypticus, Eisenia andrei*, and *Eisenia fetida* have recently been used for the effect assessment of TRWPs alone or in mixtures with other substances, often showing ambiguous results [64,68,96,97].

Studies conducted on *E. crypticus* exposed to TRWPs have shown both a survival and reproduction decrease, but it is not yet clear whether this decrease is dose-dependent or not. In Ding et al.’s study [97], exposure to TRWPs ≥ 240 mg/kg significantly reduced enchytraeid survival, while exposure at concentrations ≥ 48 mg/kg reduced their reproductive rates, showing dose-dependent toxicity; on the contrary, in Selonen et al.’s study [68], the reproduction rates of enchytraeids decreased only at the lowest (200 mg/kg) and highest (15,000 mg/kg) concentrations, showing a non-dose-dependent trend. Maybe, the different results depend on the size of particles, the chemical conditions of the soil, such as the pH, or the type of tire used. Future studies will help us to better understand these results.

Exposure to TRWPs tests conducted on the Lumbricidae family, such as *E. andrei* and *E. fetida*, did not show any mortality or effects on reproduction or avoidance behavior, while the activity of some oxidative stress biomarkers, such as ROS, GST, and CAT, showed variable trends during the exposure time [63,96,97]. The biochemical response of antioxidant enzymes, such as CAT, or secondary detoxification systems, such as GST, represent important early defense systems that different organisms activate against any disturbing factor; the increase in reactive oxygen species (ROS), on the contrary, underlines a strong condition of oxidative stress usually caused by a general breakdown of the biochemical defense systems [63]. Even if considered early defense systems, molecular biomarkers can warn about the effects that a contaminant or any stressor can have both at an individual level and at a population level, such as an effect of an endocrine disruptor, the consequences of which can affect the entire dynamics of a population species.

Earthworms have also been shown to ingest tire particles, modifying their surface and favoring the release of Zn in the digestive tract, emphasizing, once again, how the particles play a crucial role as leachate [97]. It is probable that, due to the morphological simplicity of the digestive tract, the retention time of these particles is short [101]; however, ingestion can alter gut microbial diversity, which could the change physiology and resistance to stress over time [97]. 

These results suggest that the accumulation pathways and toxic effects of these TRWPs are not yet fully understood in earthworms, which requires the implementation of standard, replicable, and more ecologically realistic methods that consider the concentrations of TRWPs to use, their size, their vehicular origin, and the effects of long-term exposure, also at the microbial level. 

### 5.2. Nematodes

In the literature, the only study conducted on the nematode *Caenorhabditis elegans* has shown that the toxicity of TRWPs may depend on the exposure time and the aging period of the contaminated soils, altering survival, growth, and brood [95]. In short-term tests on *C. elegans*, it was observed that aged soils (soil incubated with TRWPs for 30 and 75 days) showed effects on growth and brood size at 1 mg/kg d.w., while the same effects in soils not incubated with TRWPs occur at 100 mg/kg and 10,000 mg/kg, respectively. Similarly, long-term exposure tests showed early effects on survival already by the 6th day (10,000 mg/kg) in aged soils, rather than by the 8th day (10 mg/kg) in unaged soils. The authors suggest that the increase in toxicity in aged soils depends on an increase in the leachate of chemicals. Among these substances, the concentrations of metals leached into interstitial waters may not show a significant variation between treatments, demonstrating that the presence of HMs may not be the main cause of the high measured toxicity; conversely, PAHs or other organic chemical compounds may be mainly responsible for the measured toxic effects, but these studies require further investigation [95]. For the authors, it is not to be excluded that these values may depend on the low origin concentration of metals in the tire tread, too high soil pH, or too short an exposure period [95]. 

### 5.3. Springtails

Springtails such as *Folsomia candida* are often used in ecotoxicological tests related to soil contamination, with them being organisms of high ecological value and easy to grow in laboratory conditions.

In the literature, exposure to TRWPs in *F. candida* has shown different effects. In Selonen et al.’s study [68], nominal exposure concentrations of only tire particles from 0 to 15,000 mg/kg d.w. showed a decreasing trend in survival and reproduction, but not significantly. In Kim et al.’s study [62], on the other hand, TRWPs appear to reduce the growth rate in springtails at the concentration of 10,000 mg/kg d.w., probably due to the engulfment of soil pores, which stimulates springtails to expend more energy in movement or to expel ingested particles; furthermore, reproduction rates appeared to decrease in TRWP treatments, albeit not in a statistically significant way. Springtails have also been shown to ingest them, showing the potential role of the particles’ toxicity.

TRWPs can also modify the bioavailability for edaphic organisms of other contaminants; in springtails, it was demonstrated that TRWPs decrease the toxicity of insecticide chlorpyrifos, reducing its lethality (but not significantly) and its reproduction effects according to the sigmoidal concentration–response [98]. These results may be related to a seizure of chlorpyrifos by the tire particles, probably due to their lipophilicity; future tests could focus on mixture studies involving organic chemicals or metals in order to understand the mechanisms of seizure or release by TRWPs under different environmental conditions for a real risk assessment.

### 5.4. Woodlice

Woodlice are important edaphic organisms in litter and they are used as test species in studies of ecotoxicity and ecophysiology [102,103].

For springtails, in Selonen et al.’s study [98], the effect of mixing chlorpyrifos with low and high concentrations of tire particles reduced mortality and the interference on acetylcholinesterase (AchE) in *Porcellio scaber*, demonstrating reduced bioavailability of chlorpyrifos by TRWPs. AChE, an enzyme involved in neurotransmission in the cholinergic nervous system, is a target for specific and nonspecific neuro-inhibitors, such as organic and inorganic compounds; as Zn and BT are present in high concentrations, they are likely to inhibit AChE activity [98].

Other preliminary studies conducted on *P. scaber* exposed to TRWPs suggest that these induce the activation of immune-related genes in hemocytes and the hepatopancreas, modulating the immune system and the types of hemocytes in the hemolymph; in Dolar et al.’s study [99] increased ETS activity and impaired differential hemocyte count (DHC) were observed at the maximum concentration of 300 mg/kg, but hemocyte alteration was restored after the 7th day of exposure, showing immune recovery in the woodlice.

### 5.5. Plants

TRWPs have been shown to negatively affect plant growth by inducing an alteration in photosynthetic activity and polyphenolic composition, modifying soil pH, and changing litter respiration and decomposition processes [62,100]. 

The seeds of *Allium porrum*, once sterilized (0.5% solution of NaClO-bleach for 10 min and 70% ethanol for 40 s) and germinated in soils contaminated by TRWPs, in a range of 0–160,000 mg/kg according to a progression per step of 10,000 mg/kg, showed a reduction in leaf and root growth already at concentrations of 10,000 mg/kg, with this stabilizing at around 60,000 mg/kg [100]. Litter decomposition, consisting of tea leaf sachets (Lipton Green Tea, Sencha Exclusive Selection), slightly increased at low concentrations of TRWPs, but reversed its trend at concentrations > 60,000 mg/kg, decreasing slightly, underlying how the presence of TRWPs profoundly alters biogeochemical cycles and soil decomposition rates [100]. Furthermore, the soil respiration rate, as well as the pH and leached Zn levels, continued to increase until the end of the test [100]. The increase in leached Zn seems to be one of the main disturbing elements for plant growth, but its bioavailability is influenced by abiotic parameters, such as pH; in fact, heavy metals are easily absorbed at an acidic pH, while their absorption is already inhibited at more alkaline pHs. It is therefore important to know the abiotic conditions of soil to understand how TRWP contamination affects soil processes and plant homeostasis.

Similar effects were observed in the leaf and shoot growth of *Vigna radiata* exposed to TRWPs from three different vehicles (car, bike, and e-scooter) under dry soil concentrations of 1 and 10 g/kg for 28 days [62]. Specifically, TRWPs from different vehicles have been observed to alter plant homeostasis differently; bikes and e-scooters in fact reduce leaf and sprout growth, as well as altering polyphenolic biomarkers, reducing anthocyanin and flavonoid levels, and increasing nitrogen balance [62]. In addition, the photosynthetic activity of some parameters of photosystem II (PSII) was altered differently according to the TRWPs: the steady-state PSII efficacy (QYLss), the photochemical hardening coefficient (qP), and the fraction of open PSII centers (qL) were reduced in *V. radiata* leaves exposed to TRWPs from cars and bikes, while they increased in those exposed to TRWPs from e-scooters [62]. These three parameters are related to the photosynthesis of PSII and generally decrease when the photosynthetic activity of chlorophyll-a in PSII is inhibited; instead, polyphenolic compounds, such as anthocyanins and flavonoids, protect plants from reactive oxygen species by neutralizing cellular ROS levels. The decrease in anthocyanin and flavonoid levels, as well as the reduction in the photosynthetic activity of PSII, demonstrates that TRWPs induce oxidative stress in plants and that this stress factor depends on the type of tire compound, considering that tire particles emitted by different vehicles show different effects. This underlines how even the composition of the tread can influence the expected effects on plants.

## 6. Preliminary Risk Assessment and Future Projections for Urban Soils: The ‘Milan Case’

For estimating a preliminary risk related to TRWPs and co-formulates, a preliminary risk assessment was applied to a green area as an example in the city of Milan, Italy: Forlanini Park. This park is a huge Milanese urban green area located close to Forlanini Avenue, a long speedway that connects the city to the nearest Linate airport; therefore, being a busy road, TRWPs and associated contaminants could potentially be released into the adjacent Forlanini Park, which was chosen in this review as a reference area to assess the preliminary risk associated with TRWPs transport.

Parco Forlanini was also selected as a green area in Milan subject to reforestation by the ForestaMi Project, promoted by the Municipality of Milan and Co., which involves the planting of 3 million trees by 2030 in all green areas of Milan in order to purify the air, improve living conditions in the wider area, and mitigate the effects of climate change; consequently, Parco Forlanini can be considered as an outdoor laboratory for monitoring the impact of contaminants, such as TRWPs, and the effects of climate change.

In order to estimate the environmental risk of TRWPs and co-formulates, the risk quotient (RQ) was calculated by the ratio of the predicted environmental concentration (PEC) and the predicted no effect concentration (PNEC):

RQ = PEC/PNEC,(1)

Consequently, all RQ > 1 values were considered as an unacceptable risk factor, while RQ < 1 values were considered as acceptable risk factors. For evaluating the PEC, an estimation method based on the ratios between the emission factors (EFs) of tire particles or associated compounds and urban soil mass was applied:

PEC_x_ = EF_x_/M_x_, (2)

PEC_x_ = predicted environmental concentration of the x contaminant
EF_x_ = mg of the emission factor of the x contaminant emitted by registered cars
M = kilograms of urban soil considered.

### 6.1. TRWP Emission Factors

For quantifying the emissions factors, three principal methods have been developed in the literature [13,35,100]. The most widely used method for estimating EF_TRWPs_ and considered in this review combines the emission factors of tire wear by vehicle type, the number of vehicles on the road, and the annual kilometers driven [13,35,104]:

EF_TRWPs_ = EF_V_ × N_V_ × d, (3)

EF_TRWPs_ = emission factors of TRWPs (mg x vehicles/y);
EF_v_ = vehicle-specific emission factors of TRWPs (mg/km);
N_v_ = number of vehicles on the road
d = distance in km/y

The tire wear EFs by vehicle category and track type are shown in Table 4 [13,35,104]. 

To estimate the EFs of TRWPs in Forlanini Park, only the emissions from cars were considered. Considering a Milanese car fleet of 688,223 since 2020, an emission factor of cars equal to 132 mg/km [13,35,104], and the length of Forlanini Avenue of approximately 3 km, the EF_FOR_ is equal to:

EF_FOR_ = (EF_CAR_) × (N_V_) × (l_FOR_) = (1.32 × 10^2^ mg/km) × (688,223) × (3 km) = 27.2 × 10^7^ mg, (4)

**Figure 4:**
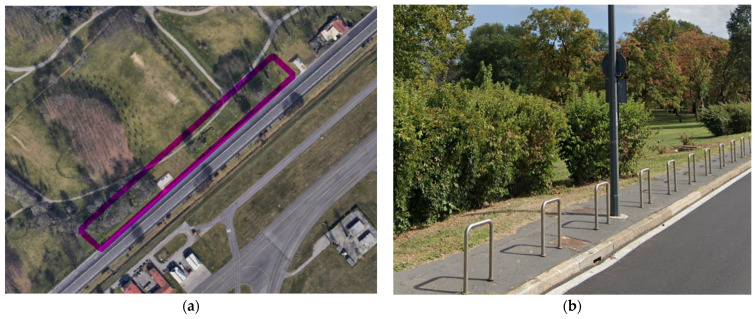
Satellite image of Forlanini Park; the portion of land considered in this review is marked in purple (**a**). Image of Forlanini Avenue, where it is possible to notice the proximity without obstacles between the road and the park (**b**).

EF_FOR_ = Maximum emission of TRWPs produced by car fleet in 3 km of Forlanini Avenue
EF_CAR_ = Estimated emission factor of TRWPs per kilometer (mg/km) from a common car (Table 4)
V_MI_ = Milan’s registered cars
l_FOR_ = Forlanini Avenue’s length (3 km) adjacent to the park (Figure 4a).

### 6.2. Mass of Considered Soil

To evaluate the PEC_TRWPs_ from Equation (2), the soil’s volume to Forlanini Park of soil susceptible to contamination by TRWPs was estimated, since this is potentially limited to the first 30 m from the roadside and to the first 10 cm of depth [66,69]; estimating that Forlanini Avenue (Figure 4) is about 3 km long, the volume of land affected by TRWPs is equal to:

V_SOIL_ = l_FOR_ × w_FOR_ × d_FOR_ = (3000 m) × (30 m) × (0.1 m) = 9.0 × 10^3^ m^3^, (5)

V_SOIL_ = Total volume of Forlanini Park soil potentially impacted by TRWPs
l_FOR_ = Forlanini Avenue’s length adjacent to the park
w_FOR_ = Width of soil impacted by TWRP transport [66,69]
d_FOR_ = Depth of soil potentially contaminated by TRWPs [66]

Estimating a general soil density of about ρ = 1.4 × 10^3^ kg/m^3^, the mass of soil volume affected by TRWPs is equal to:

M_soil_ = (ρ) × (V_soil_) = (1.4 × 10^3^ kg/m^3^) × (9.0 × 10^3^ m^3^) = 1.26 × 10^7^ kg d.w., (6)

M_soil_ = kg d.w. of the Forlanini soil mass considered
ρ = kg/m^3^ of the soil’s density
V_SOIL_ = m^3^ of the total volume of Forlanini Park’s soil from Equation (5)

### 6.3. PEC of TRWPs

By relating the number of maximum emissions of TRWPs produced by car fleets within 3 km of Forlanini Avenue to the mass of the soil potentially subject to such contamination in Forlanini Park, PEC_TRWPs_ was obtained by Equation (2):

PEC_TRWPs_ = (EF3_MI_)/(M_soil_) = (27.2 × 10^7^ mg)/(1.26 × 10^7^ kg) = 21.62 mg/kg d.w. (7)

In addition, the PECs relating to some of the most common contaminants traced in the TRWPs (organic chemicals and metals) were calculated through a proportion, using the average tire particle concentrations known in the literature (Table 5) [23,43,68,97,105,106,107,108].

The PNEC values related to the compounds have been reported in the literature (ECHA website; EU website, ref. [105]) and the PNEC for the TRWPs was determined starting from the most conservative data found in the literature (10,000 mg/kg on growth in *F. candida*) [62] and applying an assessment factor (AF) of 1000, as reported by the ECHA guidelines. It was not possible to determine the PNEC of some co-formulas, due to the paucity of data in the literature on the toxic effects of these substances in soil. 

Finally, the RQs related to the TRWPs and co-formulates were determined by Equation (1).

## 7. Discussion

The results showed an unacceptably high risk for TRWPs (RQ = 2.16) and BTs (RQ = 3.58). A low risk emerges for all the priority PAHs, from which it is also possible to identify whether the soil is heavily or slightly contaminated by summing the estimated environmental concentrations of them [109]; according to the results obtained in this review, ∑PEC_PAHs_ is equivalent to 0.115 mg/kg, which would correspond to Forlanini Park’s soil not being considered as contaminated by PAHs coming from the TRWPs of cars. Regarding HMs, a high risk did not emerge, but it should be remembered that these results can be influenced and modified by important chemical parameters, such as pH; it was not possible to define an RQ for important HMs such as Fe and Al due to the absence of soil PNEC data, which will require further investigation in the future. Despite these results, on the one hand, the tire particles seem to induce a risk related to the particles and the effects of the mixture, whereas on the other hand, they could reduce the bioavailability of some co-formulates [98]. Further investigations will help us to understand these aspects and fill some gaps in the literature.

It should be noted that the RQs evaluated for Forlanini Park, as well as any value reported in this review, are modeling results and do not take into account the real environmental contamination in that park by these TRWPs. The entire vehicle fleet of the city of Milan was also considered, as there are no data relating to the actual vehicular circulation in Forlanini Avenue, and, consequently, the value of TRWPs emitted could be overestimated; furthermore, only tire particles emitted by cars were evaluated in this review, thus underestimating the real PEC of TRWPs in Forlanini Park. In addition, the EFs and mileage depend on local factors, such as the climate conditions, type of road, driving speed, the type of vehicle, and the weight of them [110]; regarding the last one, it has been demonstrated that heavy cars increase the friction with the asphalt and, as a result, purportedly greener and heavy electric and hybrid cars release TRWPs in the same way as other petrol-powered heavy cars. This aspect should not be underestimated for functional ecological transition, and a thorough investigation is suggested for the future. 

However, this review could offer an initial and potential corpus of data for a risk assessment of TRWPs in urban soils. Actually, there is not a proper risk characterization for TRWPs contamination in soils yet. On the one hand, this lack depends on historical factors, because the attention paid to tire particle contamination is relatively recent and has mainly affected aquatic environments rather than the soil; on the other hand, this lack depends on technical and replicable factors, such as the sampling areas or type of toxicity tests, which have shown fluctuating results depending on exposure times, particle size, or model organisms.

Moreover, the data in the literature may underestimate the true contamination of TRWPs depending on the type of environmental marker used; in fact, although products of thermal degradation of SBR appear to be the most specific markers, most of the semiquantitative information in the literature on the accumulation in roadside soils is derived from Zn content measurements [35], which is a generic marker and could return underestimated vertical transport values [16,34,66,84]. The choice of environmental markers is of great importance, as it could affect the actual distribution of TRWPs in the environment, and, consequently, future studies of these estimates should be investigated using more reliable markers.

## 8. Sustainability and Risk Mitigation Measures

Risk mitigation measures (RMMs) can be proposed to reduce the impact of TRWPs and associated chemicals. As suggested by Kumar et al. [111], potential risk mitigation measures (RMMs) to reduce TRWP pollution could be contamination source reduction, environmental fate capture, and end-of-sewage treatment; most of these aspects, however, have so far been dealt with only for aquatic environments, both sewage and freshwater [35,112,113]. Hence, an accurate future study is necessary for characterization of the risk to the soil ecosystem. 

An initial intervention could be to act at the source level, promoting technologies to reduce and minimize the friction between tires and roads or promoting less use of heavy vehicles, which would help to increase the contact between the tread and the asphalt and, consequently, the release of TRWPs. Further attention could be placed on the distance of highways or roads from the soil ecosystem, increasing the width of sidewalks or, in general, reducing the contact between the soil and the road as much as possible; it is also possible to think of fairly high structures capable of blocking and retaining these rubber particles, preventing them from dispersing into the surrounding soil. However, such research has not been taken into consideration yet. 

One RMM could also be formulating new sustainable tires; in fact, tires alone contribute to 20–30% of pollution from the automotive industry [114] and many of the petrochemical components, such as synthetic rubber polymers, carbon black, and adjuvants, are not renewable [115]. Several alternative solutions regarding tire compounds have already been proposed, such as latex from guayule (*Parthenium argentatum*), as alternative and sustainable sources of polyisoprene [116] and recycled plastic bottles, as sources of reinforcing fabrics such as polyethylene terephthalate (PET) [117]. Fillers such as carbon black and silica can be obtained more sustainably through the recycling of used tires in the first case or from rice husk silica in the second case [118]. Vegetable oils, such as soybean, flax, and guayule, could replace adjuvant petrogenic oils, such as naphthenic and paraffinic oils, giving tires greater performance and making them more sustainable, with them being biodegradable and safer oils [115,119].

## 9. Conclusions

This review has analyzed different aspects of TRWPs, focusing on their chemical composition, their environmental fate, their detection techniques, their toxicity, and the associated risk, taking as an example the case study of Forlanini Park. It was found that TRWPs and BTs could constitute a high risk, which will require further confirmation through field studies. These data reinforce those reported in the literature about the potential hazard of TRWPs and associated chemicals, placing more attention on soil ecosystems and particle contamination, as well as leachate. 

In conclusion, we can define these points as future goals of the research and understanding of TRWPs contamination in soil:

(a) Greater and deeper attention to soil contamination by TRWPs;
(b) Standardization in the detection of these contaminants, with greater awareness of the choice of environmental markers;
(c) Standardization in toxicity tests, using efficient model organisms sensitive to TRWPs;
(d) Analyze the effects determined not only by the leachate but also by the particles to understand the environmental toxicity of TRWPs;
(e) Carry out interventions at the urban level to reduce both the contact between the tire tread and the asphalt and between the road and the surrounding soil;
(f) Promoting new sustainable tires as an efficient strategy to reduce TRWPs.

## Figures and Tables

**Table 1:** Concentrations of TRWPs in different environmental compartments and relative references.

Compartments	[TRWPs]	References
Road face	0.7–210 g/kg ^1^	[60,61,62,63]
Soil	0.6–117 g/kg ^1^	[62,64,65,66]
Sediment	0.3–155 g/kg ^1^	[16,34,60,63]
Sewage	0.3–179 mg/L	[34,60,61,63,71]
Freshwater	0.09–6.4 mg/L	[34,61,72,73]
Air	0.4–11 µg/m^3^	[61,62,64,66,68,69,70]

^1^ dry soil.

**Table 2:** Summary table of the most used markers for the detection of tire particles in the environment, their specificity, and relative literature references.

Markers	Methods	Specificity	References
SBR	Thermal extraction desorption gas chromatography–mass spectrometry (TED-GC/MS) Fourier transform infrared spectroscopy (FTIR) Raman spectroscopy	High	[25,34,62,65,66,69]
BTs	Thermal extraction desorption gas chromatography–mass spectrometry (TED-GC/MS)	Medium	[34,60,61,70,72,78]
Zn	Inductively coupled plasma–optical emission spectroscopy (ICP-OES)	Medium/low	[16,34,79,80,81,82,83]

**Table 3:** Summary table of the main soil organisms exposed to TRWPs. For each experiment, the dimensions of the particles, the duration of exposure, the type of soil used, and the physical parameters were kept constant, and the analyses carried out are specified. N.A.: data not available.

Species		TRWPs’ Average Size (μm)	[TRWPs’] Range (mg/kd d.w.)	Exposition Time (Days)	Soil	Physical Parameters	Analyses	Ref.
Edaphic fauna	*C. elegans*	125 μm	1–10.000 mg/kg	Acute test: 2 days Chronic test: 12 days	Texture: sand 93.3%, silt 5.0%, and clay 1.7% pH: 6.7 ± 0.2	Temperature: 20 ± 2 °C WHC: 0.34 ± 0.10 mL/g Photoperiod: in the dark	Endpoint (%): Survival rate, body length, and brood size Chemical analysis: ICP-OES	[95]
	*E. andrei*	<600 µm	1–1000 mg/kg	Acute test: 48 h Chronic test: 28 days	Texture: fine sand 95.5%, silt and clay 4.5% Organic Carbon: 2.5% pH: N.A.	Temperature: 20 ± 2 °C WHC: 50% Photoperiod: N.A.	Endpoint (%): Net response (avoidance test) and brood size Biochemical parameters: Protein content, ROS, GST, CAT, GR, AChE, CES, GPx, and MXR	[96]
	*E. fetida*	606.25 μm	10,000–200,000 mg/kg	Chronic test: 14–28 days	Standard soil (OECD 220 guideline): kaolinite clay 20%, quartz sand 70%, and sphagnum peat 10% Organic carbon: 5% Nitrogen: 0.098% pH: 6.9	Temperature: 20 ± 2 °C WHC: 25% Photoperiod: 12:12 h	Biochemical parameters: SOD, CAT, POD, GSH, and MDA Chemical analysis: ICP-MS, ATR-FTIR, and SEM	[65]
	*E. crypticus*	443.25 μm	48–30,000 mg/kg	Chronic test: 21 days	LUFA 2.2 soil: loamy sand Organic carbon: 1.73% Nitrogen: 0.19% pH: 5.6–4.94	Temperature: 20 °C WHC: 50–60% Photoperiod: 16:8 h	Endpoint (%): Survival, brood size, and gut microbial alteration Chemical analysis: GC-MS and ICP-MS	[68,97]
	*F. candida*	382.5 µm	200–15,000 mg/kg	Chronic test: 28 days	LUFA 2.2 soil: loamy sand Organic carbon: 1.73% Nitrogen: 0.19% pH: 5.6–4.94	Temperature: 20 °C WHC: 50% Photoperiod: 16:8 h	Endpoint (%): Survival, body length, and brood size	[62,68,98]
	*P. scaber*	141.45 μm	200–15,000 mg/kg	Chronic test: 21 days	LUFA 2.2 soil: loamy sand Organic carbon: 1.73% Nitrogen: 0.19% pH: 5.6–4.94	Temperature: 20 °C WHC: 40% Photoperiod: in the dark	Biochemical analysis: AChE and ETS Genetic analysis: Expression of immune-related genes in hemocytes and the digestive gland and hepatopancreas	[98,99]
Plants	*A. porrum*	125 μm	10,000–160,000 mg/kg	Chronic test: 42 days	Albic Luvisol: loamy sand Organic carbon: 1.87% Nitrogen: 0.12% pH: 5.41	Temperature: 22–18 °C WHC: 40% Photoperiod: 12:12 h	Endpoint (%): Effects on plant growth, change soil pH, and alteration in litter decomposition and respiration	[100]
	*V. radiata*	326.5 μm	1–10 g/kg	Chronic test: 28 days	Loamy sand organic matter (SOM): 0.9% pH: 5.4	Temperature: 26 °C WHC: 80% Photoperiod: 16:8 h	Endpoint (%): Growth rate of the shoots and leaves and root length Biochemical analyses: Content of polyphenolic compounds (anthocyanins, chlorophyll, flavonoids, and nitrogen balance index), and photosynthetic activities	[62]

**Table 4:** Tire wear emission factors (mg/km) of some different vehicles, depending on the type of road (urban, rural, or highway) [13,35,104].

Vehicles	EF_TRWPs_ (mg/km)		
	Urban	Rural	Highway
Car	1.32 × 10^2^	8.5 × 10^1^	1.0 × 10^2^
Bus	4.2 × 10^2^	2.7 × 10^2^	3.3 × 10^2^
Motorcycle	6.0 × 10^1^	3.9 × 10^1^	4.7 × 10^1^
Truck	6.6 × 10^2^	4.2 × 10^2^	5.2 × 10^2^
Lorry	8.5 × 10^2^	5.5 × 10^2^	6.7 × 10^2^

**Table 5:** PNEC (mg/kg d.w.), PEC (mg/kg d.w.), and RQ related to the car tire particles and some co-formulates estimated based on the concentrations reported in the literature [23,43,68,97,105,106,107,108] and potentially released in the first 30 m of topsoil of Forlanini Park, Milan. (*) = high risk; (-) = no data.

Substances		PNEC_SOIL_ [mg/kg d.w.]	PEC_SOIL_ [mg/kg d.w.]	RQ
Tire Particles		10 ^4^	21.62	2.16 *
Organic Chemicals (non-PAHs)	Benzothiazole	0.017 ^2^	0.060	3.58 *
	1-indanone	-	0.002	-
	1-octanethiol	-	0.0008	-
Organic Chemicals (PAHs)	Pyrene	1 ^2^	0.037	0.037
	Fluoranthene	1.5 ^2^	0.018	0.012
	Phenanthrene	1.8 ^2^	0.009	0.005
	Benzo[ghi]perylene	0.17 ^2^	0.004	0.024
	Anthracene	0.13 ^2^	0.002	0.022
	Acenaphthylene	0.29 ^2^	0.0004	0.001
	Benzo(b)fluoranthene	0.28 ^2^	0.006	0.022
	Chrysene	0.55 ^2^	0.012	0.023
	Indeno(1.2.3-cd)pyrene	0.13 ^2^	0.002	0.022
	Fluorene	1 ^2^	0.004	0.004
	Naphthalene	1 ^2^	0.0002	0.0002
	Benzo[a]anthracene	0.079 ^2^	0.008	0.105
	Benzo(a)pyrene	0.053 ^2^	0.004	0.075
	Acenaphthene	0.038 ^2^	0.0001	0.005
	Benzo(k)fluoranthene	0.27 ^2^	0.001	0.007
	Dibenzo(a.h)anthracene	0.054 ^2^	0.001	0.034
Metals	Zn	83.1 ^1^	19.62	0.23
	Fe	-	0.37	-
	Al	-	0.76	-
	Cu	65 ^1,3^	0.42	0.006
	Cr	21.1 ^1^	0.06	0.003
	Ni	29.9 ^1^	0.06	0.002
	Pb	212 ^1^	0.06	0.0003
	Hg	0.022 ^1^	0.0003	0.018
	Cd	0.9 ^1^	0.001	0.002

^1^ ECHA website; ^2^ EU website; ^3^ ref. [105]; ^4^ From this review.
